# Intensive group-based cognitive therapy in patients with cardiac disease and psychological distress—a randomized controlled trial protocol

**DOI:** 10.1186/s13063-021-05405-3

**Published:** 2021-07-16

**Authors:** Annette Holdgaard, Christine Eckhardt-Hansen, Thomas Lund, Christina Funch Lassen, Kirstine Lærum Sibiliz, Dan Eik Høfsten, Eva Prescott, Hanne Kruuse Rasmusen

**Affiliations:** 1 Department of Cardiology, Bispebjerg-Frederiksberg Hospital, Bispebjerg Bakke 23, bygning 67, 2400 Copenhagen, NV Denmark; 2 Department of Social Medicine, Bispebjerg-Frederiksberg Hospital, University of Copenhagen, Copenhagen, Denmark; 3 Department of Cardiology, Copenhagen University Hospital of Rigshospitalet, Copenhagen, Denmark

**Keywords:** Coronary heart disease, Cardiac rehabilitation, HADS, Depression, Anxiety, Psychological distress, Cognitive therapy

## Abstract

**Background:**

Many patients with coronary artery disease (CAD) and valvular heart disease (VHD) suffer from psychological distress. Such stress is associated with increased morbidity, reduced quality of life and delayed return to work. European guidelines emphasize recognition and intervention, but evidence-based treatment options are limited and perceived as costly. The present study will test the effect of brief, group-based cognitive therapy as an adjunct to usual cardiac rehabilitation in a randomized design.

**Methods:**

A total of 148 patients with CAD and/or VHD after surgical intervention and concomitant psychological distress (defined as HADS anxiety (A) or depression (D) score ≥8) will be randomized to either usual out-patient cardiac rehabilitation (CR) comprising an 8-week multidisciplinary programme or usual care supplemented by five group-based cognitive therapy sessions performed by trained CR nurses. A structured, standardized treatment manual will be used. Patients will be randomized 1:1 at three different sites. Additionally, a non-randomized sub-group of 40 matched patients without signs of psychological distress will be followed to investigate spontaneous variation in HADS. The primary outcome is Hospital Anxiety and Depression Score (HADS). Secondary outcomes are adherence to cardiac rehabilitation (CR), health-related quality of life measured by HeartQoL, time to return to work, adherence to lifestyle interventions and cardiovascular readmissions. Patients are followed up for 12 months.

**Discussion:**

To our knowledge, this is the first randomized controlled trial (RCT) on patients with cardiac disease with an intensive group-based programme of cognitive therapy performed by CR nurses, which makes it affordable and widely implementable. The outcome will elucidate the feasibility and effect of cognitive therapy as an adjunct to CR in patients with post-surgery CAD and/or VHD and psychological distress and could possibly benefit patients with other heart conditions as well. The clinical trial complies with the Declaration of Helsinki. The trial has been approved by The Regional Research Ethics Committee (file number H-16042832) and The Danish Data Protection Agency. The results will be disseminated as original research in peer-reviewed manuscripts.

**Trial registration:**

www.clinicaltrials.govNCT04254315. Retrospectively registered on 30 January 2020.

## Background

Psychological distress in the form of anxiety and depression is a common but often unrecognized problem in many cardiac patients. Up to 38% report psychological distress at 2 months and 33% at 12 months after myocardial infarction (MI) [1, 2]. The importance of recognizing this condition is underlined by studies that find that psychological distress not only impairs quality of life (QoL) but is also associated with increased rates of cardiac and all-cause mortality [3, 4]. Data from the national SWEDEHEART registry show that patients with persistent emotional distress following acute coronary syndrome (ACS) were more likely to die from cardiovascular and non-cardiovascular causes than those with no distress [5]. Patients undergoing valvular heart surgery have received less attention in current research, but one study has indicated that after aortic valve surgery female patients have anxiety symptoms at a level equal to that of patients with coronary artery disease (CAD) [6].

Untreated emotional distress may worsen cardiac prognosis because of behavioural mechanisms such as unhealthy lifestyle, reduced success of risk factor modification and less adherence to cardiac rehabilitation (CR) and medical treatment [7]. In one study of 5908 patients with heart disease and moderate depression, those with anxiety or stress symptoms were significantly less likely to adhere to CR than those with normal-to-mild symptoms [8]. Psychological distress may also have a direct impact on pathophysiological mechanisms such as the autonomic nervous system [9] and endothelial dysfunction [10]. Psychological distress has an impact by retaining patients with cardiac disease from returning to working. Among 20,415 patients who returned to work after MI, a quarter were no longer in employment after 1 year but were instead being supported by social benefits [11]. Important risk factors of unemployment are depression and low socioeconomic status [11, 12].

The best treatment for cardiac patients with psychological distress is uncertain, but cognitive behavioural therapy (CBT) may be an effective option. A recent systematic review and meta-analysis [13] concluded that CBT significantly reduced both depression and anxiety and improved QoL compared with controls. Group-based cognitive therapy may be a cost-effective option that can be delivered by specially trained nurses as part of a CR programme. A few studies have reported the effect of group-based CBT on psychological distress in patients with cardiac disease. In a multi-centre RCT [14], 151 unselected patients with cardiac disease received cognitively based group therapy for 12 weeks and obtained a significant additive effect on top of standard CR on a combined endpoint of stress management, cardiac biomarkers and physical activity compared with standard CR alone. Another trial with a 20-week intervention found a similar effect [15]. However, although CBT seems effective, it is still not widely implemented in CR. A main barrier is the perceived need for a comprehensive, time-consuming intervention, which is not possible in the context of a standard CR programme. Therefore, the aim of this trial is to investigate whether brief, group-based CBT integrated in CR and delivered by cardiac nurses can reduce the level of psychological distress in patients with cardiac disease.

## Methods

### Aim

The aim of the study is to develop an efficient and cost-effective model for the use of intensive group-based cognitive therapy to address psychological distress in patients with newly diagnosed CAD and/or surgically treated valvular heart disease (VHD) and concomitant psychological distress.

### Design

The study is a multi-centre, prospective, randomized clinical trial in patients with newly diagnosed CAD and/or surgically treated VHD and concomitant psychological distress defined as HADS (HADS A (Anxiety) or D (Depression) ≥ 8) and part of the work force. Patients with HADS D ≥ 11 and Beck Depression Inventory (BDI) > 17 are evaluated by a psychiatrist to ensure that patients with severe depression or other psychiatric diagnoses are not included in the study. Additionally, 40 patients with HADS score < 8 at their first visit to CR will be followed for spontaneous variation in HADS score at 3 and 6 months to determine whether screening at the first visit to CR is the optimal time at which to identify patients with psychological distress.

### Participants

The trial is a multi-centre trial planned to include 188 patients from three departments of cardiology. The main trial includes 148 randomized patients. We will also include a non-randomized subgroup of 40 matched patients (relative to inclusion and exclusion criteria) without signs of psychological distress to investigate spontaneous variation in HADS scores over time.

Inclusion criteria are:

- Referred to CR and accepting CR
- Patients with newly diagnosed CAD and/ or surgically treated VHD
- HADS score > 8 for HADS-A and/or HADS-D
- Age < 65 years
- Able to speak and understand Danish

Exclusion criteria are:

- EF < 35%
- Other serious comorbidity expected to have a serious impact on life expectancy
- Known abuse of alcohol or euphoric drugs
- Known more serious psychopathology such as schizophrenia, bipolar disorder, severe personality disorder and treatment with psychoactive drugs

### Trial procedure and randomization

Consecutive patients discharged from the hospital with newly diagnosed ischaemic heart disease and/or surgically treated VHD will be screened with HADS at their first visit to CR.

**Fig. 1:**
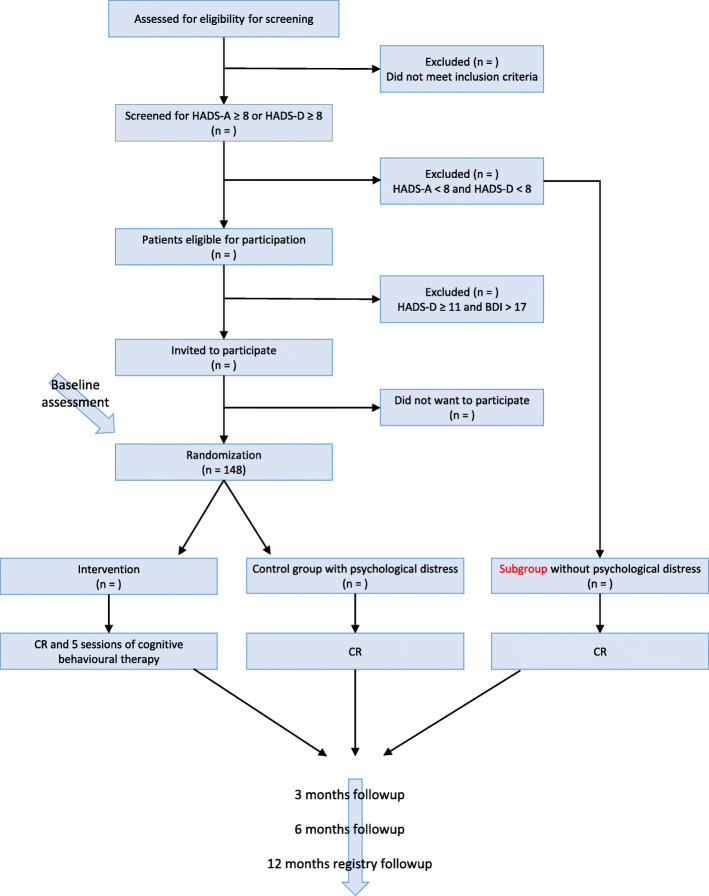
The trial diagram. Flow of patients. Participants in the intervention group follow CR and five sessions of CBT therapy and the control group follow standard CR

If a patient decides to participate in the study after having received written and verbal information from a research nurse, an informed consent form is signed and the patient is randomized to either (1) CR and intervention consisting of five sessions of CBT or (2) CR alone (Fig. 1).

Randomization is performed by an independent researcher, and the randomization programme is kept in a secured, logged hard disk. STATA II, the Ralloc programme, permuting random block sizes of 2, 4 and 6, respectively, is used for randomization. Before randomization, baseline data are collected (see Table 1).

**Table 1:** Schedule of enrolment, intervention and assessments

	Study period (months)								
	Baseline	Intervention (allocation) Group-based therapy (5 weeks)					Post-allocation (months)		Close-out
Timepoint	0	0	1 week	2 weeks	3 weeks	4 weeks	3 months	6 months	12 months
**Enrolment**									
**Eligibility screen**	x								
**Informed consent**	x								
**Allocation of patients**	x								
**Intervention**		x	x	x	x	x	x	x	
**Control**							x	x	
**Follow-up visit**							x	x	
**Assessments**									
**Background**									
Cohabitant	x						x	x	
Level of education	x								
Employment status	x						x	x	
Sick leave	x						x	x	
Return to work	x						x	x	
Index event	x								
**Clinical measurements**									
Weight	x						x	x	
Heart rate	x						x	x	
Blood pressure	x						x	x	
ECG	x						x	x	
Biochemical screening	x						x	x	
NYHA classification	x						x	x	
CCS classification	x						x	x	
**Medication**	x						x	x	
**Comorbidity**									
History of heart disease	x								
Chronic obstructive pulmonary disease	x								
Kidney disease	x								
Depression	x								
**Risk factors**									
Hypertension	x								
Dyslipidaemia	x								
Diabetes mellitus	x						x	x	
Family history	x								
Smoking	x						x	x	
Physical activity	x						x	x	
Rehabilitation received							x	x	
**Questionnaires**									
HADS	x						x	x	
HeartQol	x						x	x	
Psychosocial status	x						x	x	
**Cardiovascular readmission**									x

Both the intervention and the control group will receive usual care in interdisciplinary CR.

As our patients are still part of the work force, the follow-up visit must fit with their working schedule to reduce potential loss to follow-up. We therefore accept considerable flexibility in the scheduling of follow-up appointments. The patients receive an email or SMS as a reminder before their appointments. If they do not meet for the scheduled appointment, they will be contacted by telephone.

### SPIRIT trial schedule

This paper presents the detailed protocol for the trial “Intensive group-based cognitive therapy in patients with cardiac disease and psychological distress”. The trial is described in accordance with the current SPIRIT guidelines (Standard Protocol Items: Recommendations for Interventional Trials [16]. Results will be reported following the CONSORT (CONsolidated Standards Of Reporting Trials) guidelines for non-pharmacological interventions [17].

### Method to define psychological distress

Psychological distress in patients with cardiac disease has formerly been identified by symptoms of anxiety and depression and identified with HADS [18–23]. HADS is a 14-item questionnaire that assesses anxiety and depression levels in medically ill persons who are not admitted to psychiatric wards. The scale has two scores, HADS-A and HADS-D, and consists of seven questions to assess anxiety and seven questions to assess depression [24]. The scale is focused on psychological symptoms of mood disorders, leaving out physical symptoms that can be confused with physical illness. This is an advantage in populations with cardiac disease where symptoms such as dizziness, dyspnoea or palpitations might be related to the underlying cardiac disease and not to a potential mood disorder [25].

Psychological distress is associated with reduced QoL. Therefore, these patients’ QoL is monitored by a validated questionnaire, the HeartQoL disease-specific questionnaire. The HeartQoL measures health-related QoL in patients with heart disease. It consists of 14 items and provides two subscales: a 10-item physical subscale and a 4-item emotional subscale, which are scored from 0 to 3. Higher scores indicating better QoL [26, 27].

### Usual care

Our usual CR programme consists of a supervised 8-week outpatient exercise intervention at the hospital with 2 weekly sessions of 1.5 h with high-intensity intervals (80% of peak oxygen uptake) and resistance training. The programme is complemented with a weekly session of group-based patient education for 1.5 hours on heart disease, psychological issues and diet counselling. When needed, patients also have one or more individual sessions with a cardiologist, dietician or a nurse.

### Intervention

In addition to our usual CR programme, the intervention group will receive CBT as described below.

### Cognitive behavioural therapy (CBT) background

CBT is an established and recognized evidence-based psychotherapeutic method based on clinical experience, theory and research. Three categories of CBT exist. The first originates from behavioural therapy; the second rests on theories about thoughts and their influence on the body, emotions and actions (cognitive therapy), while the third CBT category (such as acceptance and commitment therapy (ACT), metacognitive therapy and mindfulness-based therapy) is based on acceptance of one's thoughts and feelings. In cognitive therapy, focus is on which of the patient’s thoughts and behavioural patterns may create and maintain current problems and influence functional levels. By changing thoughts and patterns of behaviour, the level of functioning and/or QoL may also be changed [28, 29]. In ACT, the focus is rather on accepting the current situation combined with a commitment to change inappropriate behaviour. In this context, focus is given to the purpose of one’s thinking rather than to the contents of a given thought. CBT and ACT have much in common; they both focus on the present and private internal experiences, and they both present the patient with skills that can be applied in different contexts. Because they share these characteristics, it is possible and even helpful to integrate the two approaches. In the present study, sessions 2, 3, and 5 are based on concepts of changing thoughts and behaviours (CBT), and sessions 1 and 4 are primarily based on ACT interventions [30].

### CBT intervention

The CBT intervention is a psycho-educational group course conducted as five sessions led by an experienced nurse under the supervision of a psychologist who is a specialist in CBT. There will be 3–4 patients in each group for the therapy sessions. Each session will last 2 h and has a set structure, with the use of homework and always ending with the opportunity to give feedback [28].

The five sessions contain:

1. Introduction to CBT and mapping of own values
2. Anxiety and anxiety reduction techniques
3. Functional analysis (awareness of consequences of own behaviour)
4. Concerns and strategies for dealing with concerns
5. Balance between requirements and forces as well as conclusion

#### First session

**Fig. 2:**
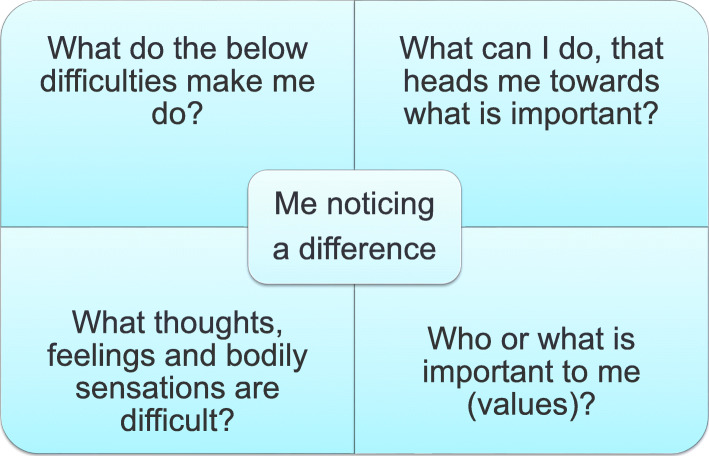
The matrix shows the significance of discovering one’s values to overcome difficulties

The experience of having a severe illness sets in motion a variety of existential thoughts about life and death and about what is important in life. Thus, the first session seeks to clarify which values the patient has and which actions bring him/her into alignment with these values. It also reveals which difficulties prevent him from acting the way he really wants to act and how a given behaviour is related to what is important to him. For example, a heart patient may have a value linked to health and the associated behaviour might be to follow the dietician’s advice and to continue exercising. His difficulties might be associated with fear of having another heart attack, which could lead to inactivity. The assigned homework in this session is completing a matrix (see Fig. 2) [31].

#### Second session

**Fig. 3:**
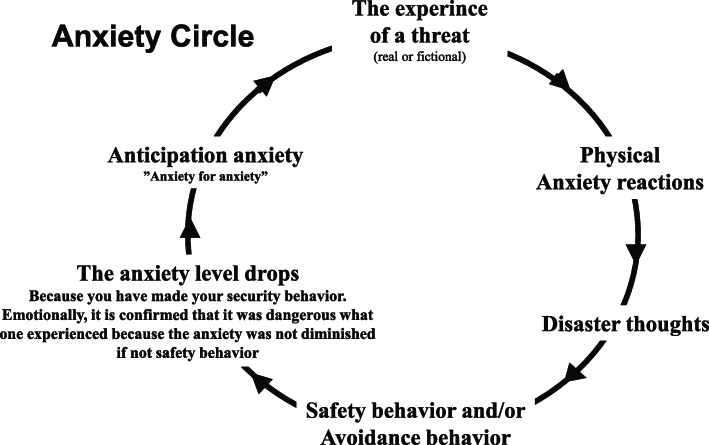
Circle of anxiety demonstrates a correlation between thoughts, feelings and behaviour. The anxiety circle is inspired by Esben Hougaard

After a heart attack, patients commonly fear another attack. The second session is thus based on anxiety. Initially, it is emphasized that anxiety is a normal feeling and a natural reaction to danger. Anxiety becomes inconvenient when connected to an “imagined” danger and/or takes on the character of an “excessive” danger. The physiology of anxiety is thoroughly reviewed, so that the patient will be able to recognize its physical components and thus distinguish between a regular heart attack and anxiety symptoms. When the basic conditions are explained in this way, the anxiety circle is reviewed; the anxiety circle describes how an experienced threat can cause physical reactions and catastrophizing thoughts. This often leads to avoidance behaviour and/or safety behaviour. Of course, when anxiety-filled situations are avoided, anxiety diminishes, but the expectation of anxiety rises, and the person may begin to fear a similar situation in the future (see Fig. 3). This session ends with the introduction of various types of strategies as well as explanations of why it is important gradually to expose oneself to situations in which it is difficult to keep anxiety at a low level. The homework for this session is to begin gradual exposure and to apply the strategies in everyday life [32].

#### Third session

**Fig. 4:**
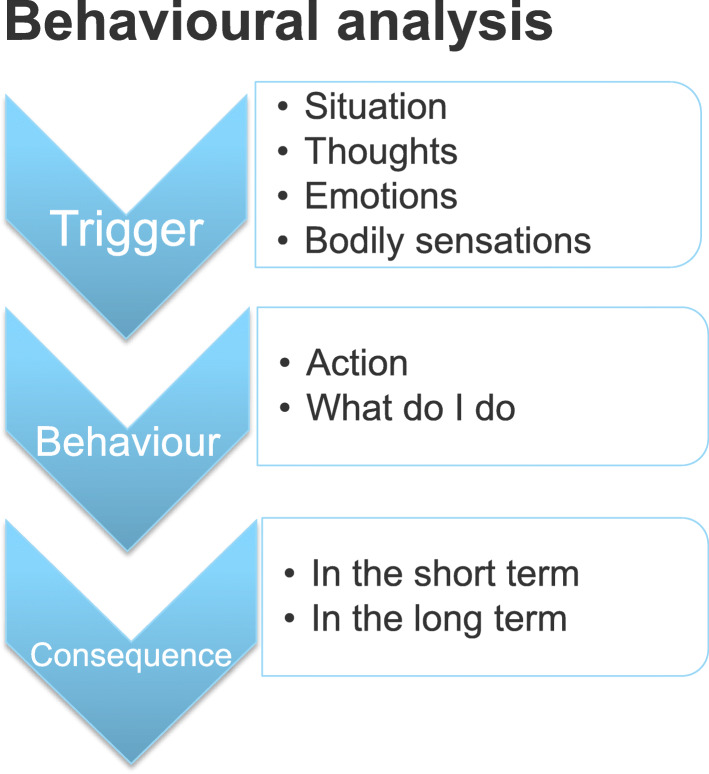
Behavioural analysis demonstrates the consequences of behaviour and what leads to the behaviour

After CAD and surgically treated VHD, several lifestyle changes are recommended, such as a change of diet and initial part-time sick leave. It can be difficult to change lifestyle and incorporate new habits. Therefore, the third session deals with the analysis and consequences of current behaviour. For example, a patient is satisfied with his work, wherefore he works 50 h per week. The consequence of this is that in the short term he is satisfied and happy while not having time to recover from his illness, which can have serious implications in the longer term. Homework in this session is the completion of a “behavioural analysis” form (see Fig. 4).

#### Fourth session

CAD and VHD give rise to many concerns that potentially limit the patient. Worry is a way of thinking that can lead to problem-solving, but it can also lead to pondering. Therefore, the fourth session is about recognizing one’s own thinking and the influence it has on one’s behaviour in order to move towards a more constructive behaviour. The session broadly explains several strategies including acceptance and diffusion (creating distance to thought). Homework is to recognize if/when one ponders/worries and if so, to possibly apply some of the strategies [33].

#### Fifth session

The last session focuses on how to make optimal use of the strengths the patient possesses. It illustrates how to balance demands and strengths with an emphasis on committing to nourishing and long-term strategies. It can for example be helpful to apply new strategies such as breaking up a larger task into smaller steps. In the latter half of this session, the matrix from the first session is explained and included more explicitly to examine whether the participants may have changed their strategies and comprehension in comparison with the beginning of the course. The session is concluded with feedback on the entire process [34, 35].

### Primary outcome

The primary outcome is mean (method of aggregation) change (metric) in psychological distress (domain) measured by HADS total score (specific measurement) from baseline to three months (time) in the intervention group compared with the usual care group. This outcome may be subject to ascertainment bias when blinding is not possible, e.g. social desirability bias causing participants to respond more favourably. We have attempted to limit this by administering the paper questionnaires to be filled in unsupervised. Furthermore, since both the intervention and the control group receive usual CR in the same cardiac settings, both groups may be subject to similar social desirability bias. At the 6-month follow-up, we anticipate that this bias will be reduced.

### Secondary outcomes

Secondary outcomes are adherence to CR defined as > 80% participation in planned training sessions (used as a secondary outcome under the hypothesis that participation in group-based therapy will improve CR adherence), health-related QoL measured by the questionnaire HeartQoL, return to work, marital status, adherence to medication and lifestyle interventions for CVD risk factor goals defined as four separate outcomes: HbA1c < 48, BP < 135/85 mmHg, BMI < 30 and LDL < 1.4 mmol/l, physical activity (days per week with ≥ 30 min of moderate physical activity) and cardiovascular readmissions at 12 months.

### Sample size and power calculation

The primary outcome is a change in HADS score at 3 months. A clinically relevant difference between groups would be a reduction in the total sum score of 2. In a previous study [36], the change within each group was normally distributed with a standard deviation (SD) of 2.5. We have here used a more conservative estimate with an SD of 4. Under these assumptions with a risk of type 1 error of 0.05 and a power of 0.8, 64 patients should be included in each group. Due to the risk of dropout of patients included in this type of trial, the total number of patients included is increased by 15% to 148 patients

STATA II, the Ralloc programme is used for randomization, permuting random block sizes of 2,4 and 6.

To assess the spontaneous variation in HADS scores over time, we will investigate a non-randomized group of 40 matched patients (relative to inclusion and exclusion criteria) without signs of psychological distress (i.e. HADS A < 8 and HADS D < 8) at first measurement with re-evaluation after 3 and 6 months. With 64 patients in the usual care arm and assuming a SD of 4 as above, we will have 80% power to detect a difference in change in HADS score (delta HADS) of 2.3 or more between these two groups.

### Data management

Data will be collected at baseline and follow-up visits. Questionnaire responses (all on paper) will be entered into electronic form in REDCap immediately after the assessment. Data from patient records (all electronic) will also be entered into REDCap. All participants will be identified by unique study identification numbers that will be included in a password-protected file. Data will be stored in accordance with the rules of the Danish Data Protection Agency. REDCap meets all criteria for the handling of patient data in accordance with the laws on the processing of personal data. The principal investigator HR, AH and EP will have unlimited access to the final trial dataset.

### Statistical analysis

The primary analysis will be a comparison of the intervention and control group in mixed-models linear regression with robust standard errors with 3-month values as outcome, and baseline and 6 months values will be used as adjustment variables. Analyses will also be adjusted for the centre and tested for treatment-centre interaction. For the analysis of the secondary outcomes return to work (yes/no), HbA1c< 48, BP< 135/85 mmHg, BMI< 30, LDL< 1.4 mmol/l and physical activity, we will also apply repeated measures regression. For adherence to CR (> 80%), we will compare groups by logistic regression adjusting for the centre. For the outcome of re-admission at 12 months, we will compare outcomes across groups with Kaplan-Meier curves and time-to-event analyses (Cox regression) adjusting for age, left ventricular ejection fraction, co-morbidities and risk factors as described above. A two-sided P < 0.05 will be considered statistically significant, and all primary outcome analyses will be performed without knowledge of group allocation.

We will compare the primary outcome in intervention and control groups with the spontaneous variation in the non-distressed subgroup of 40 participants by separate mixed-models linear regression adjusting for baseline values and centre.

The primary reported analyses will be carried out as a modified intention-to-treat as participants with missing data on a primary or secondary outcome will be excluded from the corresponding analyses. The analysis is supplemented with per-protocol analyses defined by participation in ≥ 4 of the 5 group-based sessions. Missing information on other co-variates will be permuted if there is < 20% missing data for the co-variate in question.

## Discussion

Psychological distress is often an overseen problem in patients with cardiac disease receiving CR the consequences of which are increased morbidity and mortality. If supported, our intervention will be a cost-effective and accessible way to improve QoL and reduce morbidity in these patients, and it might impact daily clinical practice. It is expected that results from the study will contribute to fill an important gap in our knowledge of how to address the physical and psychological needs of patients having undergone heart surgery.

## Trial status

The study is currently enrolling patients and was planned to complete patient enrolment in 2021. However, due to the COVID-19 pandemic, the study is now expected to be extended until 2022. Due to the lockdown in Denmark, it was not possible to include patients from 11 March 2020 until 1 June 2020. The patients randomized immediately before this period had to be excluded as the time until start of the intervention was too long.

## Data Availability

Danish legislation does not allow us to share data even if anonymized. The data will be made available in aggregated form from the authors upon request.

## Abbreviations

ACS: Acute coronary syndrome
ACT: Acceptance and commitment therapy
AHA: American Heart Association
BDI: Beck Depression Inventory
BMI: Body mass index
BP: Blood pressure
CBT: Cognitive behavioural therapy
CCS: Canadian Cardiovascular Society grading of angina pectoris
CI: Confidence interval
CONSORT: CONsolidated Standards of Reporting Trials
CR: Cardiac rehabilitation
ECG: Electrocardiogram
ESC: European Society of Cardiology
HADS: Hospital Anxiety and Depression Scale
HbA1: Glycated haemoglobin
HeartQol: Heart-related quality of life
HR: Hazard ratio
LDL: Low-density lipoprotein
NYHA: New York Heart Association
ICBT: Internet-based cognitive behavioural therapy
PCI: Percutaneous coronary intervention
Qol: Quality of life
RCT: Randomized controlled trial
SD: Standard deviation
Smartex-HF study: Controlled study of myocardial recovery after interval training in heart failure
VHD: Valvular heart disease
